# Successful Laporatomy for Intussusception of the Bowels*Read before the Georgia Medical Association, April, 1891.

**Published:** 1891-06

**Authors:** A. S. Johnson

**Affiliations:** Bowman, Ga.


### SUCCESSFUL LAPORATOMY FOR INTUSSUSCEPTION OF THE BOWELS.*

BY A. S. JOHNSON, M. D., BOWMAN, GA.

I was called to see a young man, I. S. G., age 18 years, of good parentage, was raised on the farm. He was taken sick on the 23d of August, with pain in the bowels. I was called on the night of 23d to see this young man. I diagnosed the case one of three things: Stricture, impacted feces or intussusception. I gave him three doses of chloride of mercury three hours apart. Three hours after I gave castor oil and it

*Read before the Georgia Medical Association, April, 1891.

failed to move the bowels. After this I commenced to use large enemas of warm water, frequently, all to no effect. This was done on the 23d and on the 24th; on the 25th I gave him croton oil without any relief. At this time he commenced to have severe emesis; discontinued internal remedies, had patient placed on back, knees flexed upon the abdomen, and with the strongest Davidson syringe I could get, pumped every drop of water I could get into the bowels, all to no effect. On the 27th inst. I inserted a rubber tube 10 or 12 inches long, up the rectum. Into this tube I inserted the nozzle of the syringe. After securing the nozzle of this syringe by means of a string, so as to prevent the water returning, I also used a compress. Commenced to use the water with greater force with the hope of relieving the invagination with the same results as before. I decided that it was impossible to relieve him without an operation. At 4 o'clock p. m., of the 28th, assisted by Dr. H. B. Harper, after placing the patient under an anaesthetic (chloroform) with carbolized water as a disinfectant, I made a 5-inch incision in the median line commencing one inch below the umbilicus; very little hemorrhage.

After I had opened the cavity three of my fingers were introduced, but could not locate the obstruction. I lifted the bowel out and traced them until I found the invaginated portion. It was in the upper third of the descending colon I had no trouble in reducing it; the bowels were in a high state of conjestion, also the mesentery, the color of which was a purple red. I closed the wound with six silk sutures through and through, did not wash out the cavity with anything, nor did I use any drainage tube; dressed the wound with carbolized lint. He came out from the chloroform all right—time of operation, 30 minutes.

A half hour after, he had a small evacuation of feces. One hour later, again they acted, then I gave him an injection of morphia, 1-4 grain, hypodermically. After which the vomiting ceased and he rested well the remainder of the night. Diet restricted for four or five days to rice-water entirely.

The second day after the operation there was a slight rise in the temperature. I gave him small doses of gelsemium and veratrum to control the heart's action; kept his bowels quiet for two days, after which I moved them with warm water enemas with the desired effect. He made a rapid recovery and is fully restored. It is thought that the intussusception was caused by jumping down from an elevation of some ten or fifteen feet.

